# Metabolic manipulation of methanogens for methane machinations

**DOI:** 10.1111/1751-7915.12425

**Published:** 2016-10-17

**Authors:** Thomas K. Wood

**Affiliations:** ^1^ Department of Chemical EngineeringPennsylvania State UniversityUniversity ParkPA16802‐4400USA; ^2^ Department of Biochemistry and Molecular BiologyPennsylvania State UniversityUniversity ParkPA16802‐4400USA

The U.S.A is moving toward energy independence; a distant memory are the long lines for gasoline of the 1970s and fading is the promise of competitive biofuels from *Escherichia coli* (Liu and Khosla, 2010; Steen *et al*., 2010). The reason is cheap methane. Global amounts of shale gas total 7300 trillion cubic feet (U.S. Energy Information Administration, 2013) and its major constituent is methane. This remarkable availability of methane is now driving synthetic biology, and an exciting prediction is that methane will be harnessed for biotechnological applications using not the traditional workhorse *E. coli* or aerobic methanotrophs*,* but instead, using archaeal strains, specifically methanogens, in anaerobic fermentations based on biosynthetic pathways such as that recently shown to convert methane to the biotechnological building block acetate (Soo *et al*., 2016).

As opposed to chemical plants which employ Fischer–Tropsch processes to convert methane into liquid fuels and require complex technology that demands large‐scale investment up to ~$20 billion, biological conversion of methane is a more economically and environmentally sustainable, as it requires a smaller footprint and is less technologically complex (Haynes and Gonzalez, 2014). Hence, harnessing methane has been recognized as one of the most important near‐term goals for biochemical engineering (Lee and Kim, 2015). Notably, from the recent realization that anaerobic processes confer higher energy and carbon yield efficiencies with lower CO_2_ emissions than aerobic ones for converting methane into products (Haynes and Gonzalez, 2014), there is interest in using anaerobes rather than the traditional, better‐studied aerobic methanotrophs (Lawton and Rosenzweig, 2016).

The first process used to capture methane anaerobically for biotechnology applications (Soo *et al*., 2016) is based on the natural process of anaerobic methane oxidation (AOM), which efficiently captures up to 300 Tg of methane per year to limit global methane emissions (Knittel and Boetius, 2009). AOM occurs in natural consortia consisting of anaerobic methanotrophic archaeal populations and syntrophic bacteria. Methane is activated by reversing methanogenesis and was hypothesized to be catalysed by methyl‐coenzyme M reductase (Mcr) based on the prevalence of *mcr* genes in ANME populations (Hallam *et al*., 2004) and the trace AOM seen in the anaerobic methanogens *Methanothermobacter marburgensis* (Scheller *et al*., 2010) and *Methanosarcina acetivorans* (Moran *et al*., 2005, 2007). This hypothesis had been difficult to prove as these natural consortia are enigmatic due to their long lag phase (~60 years) (Dale *et al*., 2008) and doubling time (~7 months) (Nauhaus *et al*., 2007). Critically, no one has been able to culture these organisms independently (Scheller *et al*., 2010).

Of course the way to circumvent the problem of not being able to culture anaerobic methanotrophic archaeal populations is to utilize the metagenome of these organisms from a microbial mat in the Black Sea (Meyerdierks *et al*., 2010; Shima *et al*., 2012). From this metagenome, Soo *et al*. (2016) cloned the genes encoding the Mcr (3.9 kb) and expressed this 280 kDa heterohexameric (αβγ)_2_ protein complex in the methanogenic host *M. acetivorans*. This host was chosen as it has the largest archaeal genome (Galagan *et al*., 2002), is genetically tractable (Kohler and Metcalf, 2012) and encodes a native Mcr for producing methane during methanogenesis; hence, it was reasoned that this host may be able to provide the methylthio‐F_430_ cofactor (or suitable substitute) to produce active Mcr from the anaerobic methanotrophic archaeal population. The *M. acetivorans* host also contains all the enzymes required to convert captured methane to acetate, by running methanogenesis in reverse (after methanogenic Mcr is replaced with the methanotrophic Mcr from the Black Sea). In effect, the first anaerobic organism that grows on methane as a pure culture was created. Remarkably, the engineered strain grows as a biofilm on solid ferric chloride which was reduced by the electrons generated by growth on methane (Soo *et al*., 2016). Carbonate in the medium and methane in the headspace were converted into the two carbons of acetate as shown by ^13^C labelling of both substrates (Soo *et al*., 2016). These results in effect put an end to the decade's old debate about whether methane can be fixed by running methanogenesis in reverse (Knittel and Boetius, 2009). They also will now enable Mcr to be studied biochemically as it is produced for the first time in active form.

Critically, by capturing methane anaerobically in a pure strain, a new field of biochemical engineering has been created, that of the anaerobic conversion of methane for biotechnological applications. Although not an end in itself, the acetate produced from methane by reversing methanogenesis (Soo *et al*., 2016) is a building block for many products. For example, via additional metabolic engineering and production of active methanotrophic Mcr, we have now converted methane into the pure stereoisomer *L‐*lactate which may be used in cosmetics, foods and pharmaceuticals. In addition, by combining the engineered archaeal strain that captures methane with suitable consortia, we have found that we can directly convert methane into electricity in fuel cells. This allows electricity to be generated at remote fracking sites and foregoes the expenditure of billions of dollars that are required for methane transport as well as may help limit methane emissions (methane is a potent greenhouse gas). Therefore, the future is bright for harnessing biologically the world's deposits of methane; i.e. one can envision anaerobic cell factories in which myriad products are produced from methane as well as a methane‐driven, electricity generating industry.

## Conflict of interest

The author has no conflict of interest to declare.
